# Genomic Runs of Homozygosity Record Population History and Consanguinity

**DOI:** 10.1371/journal.pone.0013996

**Published:** 2010-11-15

**Authors:** Mirna Kirin, Ruth McQuillan, Christopher S. Franklin, Harry Campbell, Paul M. McKeigue, James F. Wilson

**Affiliations:** Centre for Population Health Sciences, University of Edinburgh, Edinburgh, United Kingdom; Erasmus University Medical Center, Netherlands

## Abstract

The human genome is characterised by many runs of homozygous genotypes, where identical haplotypes were inherited from each parent. The length of each run is determined partly by the number of generations since the common ancestor: offspring of cousin marriages have long runs of homozygosity (ROH), while the numerous shorter tracts relate to shared ancestry tens and hundreds of generations ago. Human populations have experienced a wide range of demographic histories and hold diverse cultural attitudes to consanguinity. In a global population dataset, genome-wide analysis of long and shorter ROH allows categorisation of the mainly indigenous populations sampled here into four major groups in which the majority of the population are inferred to have: (a) recent parental relatedness (south and west Asians); (b) shared parental ancestry arising hundreds to thousands of years ago through long term isolation and restricted effective population size (N_e_), but little recent inbreeding (Oceanians); (c) both ancient and recent parental relatedness (Native Americans); and (d) only the background level of shared ancestry relating to continental N_e_ (predominantly urban Europeans and East Asians; lowest of all in sub-Saharan African agriculturalists), and the occasional cryptically inbred individual. Moreover, individuals can be positioned along axes representing this demographic historic space. Long runs of homozygosity are therefore a globally widespread and under-appreciated characteristic of our genomes, which record past consanguinity and population isolation and provide a distinctive record of the demographic history of an individual's ancestors. Individual ROH measures will also allow quantification of the disease risk arising from polygenic recessive effects.

## Introduction

When an individual's parents share a relatively recent common ancestor they will share large parts of their genomes that are identical-by-descent. If both of the parents transmit the same segment to the child, the child will be homozygous for that segment, thus creating a run of homozygosity (ROH)[1]. However, parents can pass on identical chromosomal segments to a child even when the relationship between them is a very distant one. There is therefore a continuum of homozygous segment length, depending on the degree of shared parental ancestry and its age. ROH due to recent inbreeding will tend to be longer, because there has been little opportunity for recombination to break up the segments that are identical-by-descent. On the other hand, ROH of much older origin are generally much shorter because the chromosomal segments have been broken down by repeated meioses. Some longer segments may, however, persist due to locally low recombination rates in certain areas of the genome. Based on this observation it is of interest to compare the extent of homozygosity between populations with different degrees of isolation and consanguinity. The availability of genome scan technology, capable of genotyping individuals at hundreds of thousands of markers has made this observational approach possible.

Studies that have focused on identifying the extent of homozygosity in individuals born into consanguineous unions (marriages between close relatives; second cousins or closer) [2], [3] found that the degree of homozygosity was higher than expected in consanguineous individuals. This emphasised the problem of estimating coefficients of inbreeding from pedigrees, which ignore more ancient familial relatedness that is not reported in genealogies. Recent studies that have focused on identifying ROH have revealed that they are very common and longer than expected in unrelated individuals from outbred populations, such as the HapMap [4], [5] or European American controls [6]. A detailed study of four populations in Europe revealed clear differentiation between endogamous communities (who tend to marry within the population) and more cosmopolitan groups in the proportion of subjects with ROH of a given length, the total number and total length of ROH [1]. The good correlation with pedigree inbreeding coefficients (*r* = 0.86; [1]) was reflected in the fact that demonstrably outbred individuals never carried ROH over 4 Mb in length, while ROH over 10 Mb were very rarely seen in cosmopolitan populations. No study has assessed genome-wide patterns of homozygosity in human populations representing the global diversity in geography and demographic history. Empirical data [1], [7] and theory [8] suggest a relationship to cultural consanguinity (typically marriage between first or second cousins), population size and degree of endogamy (within-population marriage). ROH may thus provide a new approach to illuminate human population sizes and mating patterns in the past.

## Results

**Figure 1:**
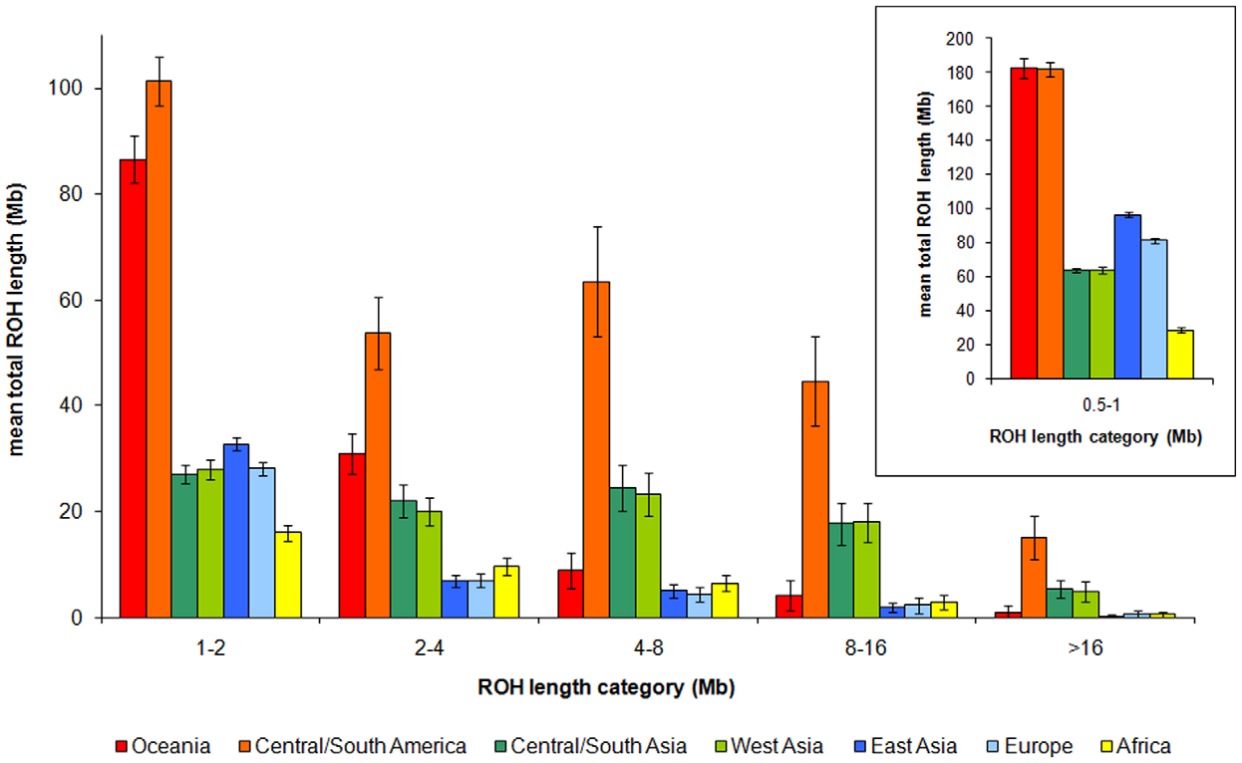
Global distribution of long runs of homozygosity. The average total length of the genome in runs of homozygosity in a number of length categories is plotted for each continental region. The data for runs under 1 Mb (inset) are plotted on a different scale. For comparison, chromosome 1 is 224 Mb in length.

To investigate the degree to which genomic ROH reflect known demographic parameters and to describe their pattern across the globe, we analysed 1043 individuals from 51 different populations collected and genotyped as part of the Human Genome Diversity Project (HGDP) [9], [10]. The proportion of the genome in ROH of different lengths (and inferred to be autozygous) varies dramatically across continents (Fig. 1). A linear mixed model, with continental regions fitted as fixed effects and populations (and individuals within populations) as random effects, was used to check the significance of differences in the length and number of ROH between all 7 geographical regions. The median, 75th percentile and number of ROH were all significantly different between the continental regions (File S1).

The Native American groups (sampled from central and south America) stand apart from all others in having the longest genomic stretches of homozygosity for all ROH length categories. The most extreme individual, a Karitiana from the Brazilian Amazon, has a total of ∼861 Mb in runs of homozygosity above 500 kb in length, equivalent to one third of the genome. 67% of Native Americans have at least one ROH longer than 10 Mb, consistent with considerable inbreeding (not shown). Samples from the Americas are also differentiated from all except the Oceanians for shorter ROH, measuring 0.5 to 2 Mb. The Oceanian populations have around three times as much of their genome in ROH of this size compared to Africans and Eurasians. The Papuan and Melanesian populations do not, however, differ from Africans and Eurasians for ROH above 8 Mb in size, indicating that the effect of reduced population size and isolation is confined to smaller ROH. The low genetic diversity in America and Oceania [10], together with the ascertainment bias resulting from greater genetic distances from the European, East Asian and sub-Saharan African SNP discovery panel populations will inflate homozygosity and therefore estimates of autozygosity in these populations. To overcome this we excluded SNPs with minor allele frequencies less than 1% in each of our 7 continental regions to create the consensus panel of SNPs used in the analyses.

South/Central Asians and West Asians have more than three times as many ROH in all categories over 4 Mb long than sub-Saharan Africans and other Eurasians. 19% of individuals from these populations have ROH over 16 Mb in length, consistent with the high prevalence of consanguineous marriage (marriage between individuals who are second cousins or closer) in these populations [11]. Neither the proportion of individuals with ROH in each length category (by χ^2^; File S1), nor the individual sum length of ROH above different length thresholds (by Mann-Whitney U; File S1) differs significantly between these two regions, confirming the overall similarity of their ROH.

Europeans and East Asians have very similar ROH profiles in all but the shortest category (0.5–1 Mb). There are no significant differences between either the percentage of individuals with ROH of different lengths or sum length of ROH above different length thresholds (>1.5 Mb) for these two continental groupings (File S1). This is not surprising because both of these groups are mainly represented here by fairly large populations with no documented preference for consanguineous marriage. Considered together, the South and West Asians on the one hand are significantly different from the East Asians and Europeans on the other, for all lengths of ROH (File S1). Short ROH are more common in East Asia, consistent with a smaller effective population size (N_e_) after the exit from Africa [12]. In accordance with their longer population history and larger N_e_, sub-Saharan Africans have the fewest short ROH. A Mandenka from Senegal has the lowest proportion of the genome in ROH above 0.5 Mb: only 25 Mb. Even in sub-Saharan Africans, however, ROH are common: all individuals have ROH measuring 1–2 Mb and a small proportion (∼3%) have very long ROH (>16 Mb).

**Figure 2:**
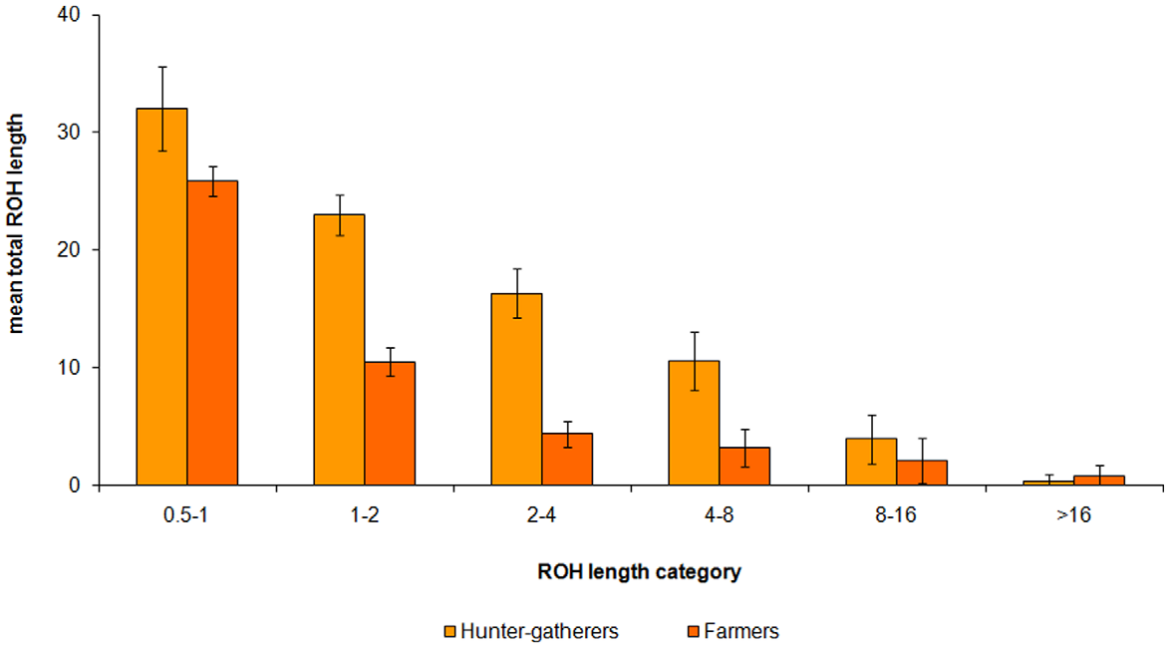
Runs of homozygosity in African hunter-gatherers and agriculturalist heritage populations. The average total length of the genome in ROH in a number of length categories is plotted.

When individual populations within continents are considered, it is possible to resolve finer-scale differences in demographic history. The sub-Saharan African populations can be divided into small hunter-gatherer and large, agriculturalist-heritage communities, which today are often urbanised. The Biaka and Mbuti pygmies and !Kung San have on average more than double the total length of ROH between 1–16 Mb compared to the Bantu, Yoruba and Mandenka (Fig 2). The large sub-Saharan African populations are very similar to Europe and East Asia for ROH longer than 2 Mb, but have less than one third the total length Europeans typically have in ROH in the smallest size category. The overall sub-Saharan African mean is inflated by the sampling of hunter-gatherer groups.

Pedigree-based estimates of the mean inbreeding coefficient, F_ped_, are available for 17 of the 51 populations and show highly significant and strong correlations with the very long ROH observed, peaking for the sum of ROH >10 Mb (*r = *0.87, *p*≤0.0001). As the proportion of the genome in ROH over 5 Mb, termed F_ROH5_ is also highly correlated with individual F_ped_ in a well characterised European population (*r = *0.86; [1]), this implies that ROH can be used to infer inbreeding in the absence of pedigree or survey data. In contrast, the sum of ROH below 5 Mb has no relationship (*r* = 0.02, *p* = 0.94) with mean F_ped_ in each population, nor with modern population size (*r* = –0.19, *p* = 0.19). However, the sum of the shortest ROH we consider here, from 0.5–1 Mb, is inversely correlated (*r* = –0.51; *p* = 0.00012) with estimates of male effective population size based on Y chromosome diversity in the same populations [13]. F_ROH_ varies enormously across the world (Table 1), reflecting the distribution of long and shorter runs. F_ROH0.5_ sums both the very long runs and the large number of shorter ROH. The estimate derived from longer runs (F_ROH5_) is considerably more variable within populations, as these longer ROH are of more recent origin and specific to individuals with consanguineous parents. We found a perfect correlation (*r* = 0.99, *p*<0.0001, Supplementary File S1) between the ROH we identify and those called using thresholds based on fine scale recombination rates in HapMap [5].

**Table 1:** Continental mean genomic inbreeding coefficients (F_ROH_).

	F_ROH_	F_ROH_	Mean (SD) sum of ROH (Mb)	
Region	>0.5	>5	>0.5	>5
Oceania	11.7%	0.41%	314 (41.9)	10.9 (15.5)
America	17.2%	3.93%	460 (171)	105 (97.3)
C/S Asia	6.0%	1.51%	160 (96.7)	40.5 (58.1)
West Asia	5.9%	1.43%	158 (85.4)	38.4 (50.6)
East Asia	5.3%	0.20%	143 (31.4)	5.5 (14.1)
Europe	4.6%	0.21%	124 (31.3)	5.5 (16.9)
Africa	2.4%	0.29%	64.0 (30.2)	7.7 (13.6)

The average %F_ROH_ and corresponding mean total length in ROH (and standard deviation) are presented using 0.5 and 5 Mb minimum ROH length thresholds.

**Figure 3:**
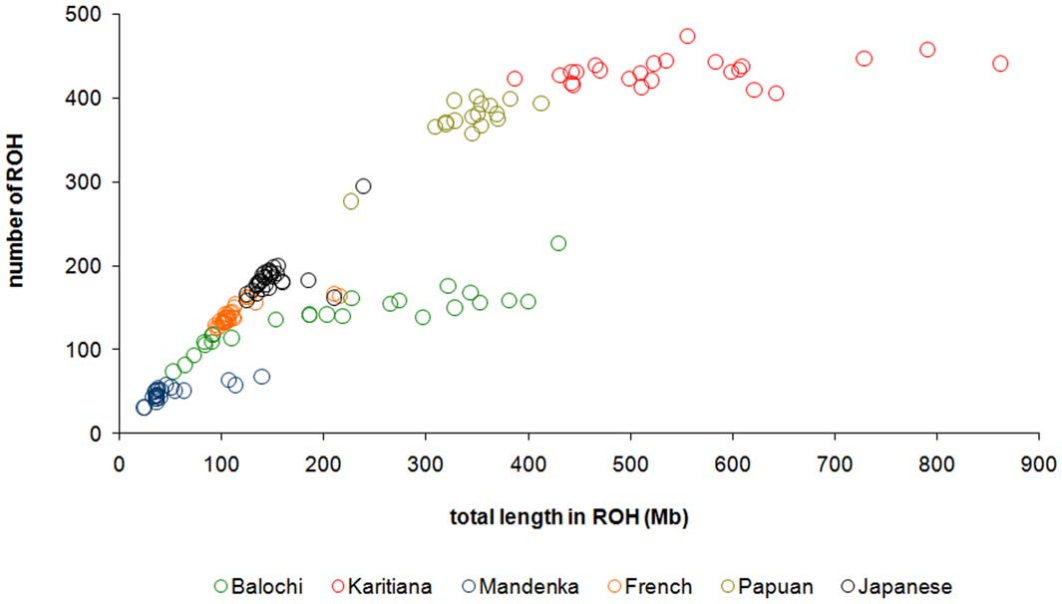
Individual patterns of long runs of homozygosity. The number of runs of homozygosity compared to the total length in ROH for individuals from a number of illustrative populations. The Karitiana are from the Brazilian Amazon, the Mandenka from Senegal and the Baluchi from Pakistan. Cryptically inbred French, Japanese and Mandenka individuals plot to the right of the main population cluster in each case, as they carry longer ROH, but not that many more ROH. The length in ROH is very variable in the consanguineous Baluchi and Karitiana populations.

The complement of ROH in an individual genome may be represented efficiently by plotting the number against the total length (representative populations are shown in Fig 3; all populations are plotted in supplementary File S1). The same trends across populations can be seen; however the variation among individuals within populations is also apparent. Individuals from most populations tend to cluster on the plot, particularly with regard to the number of ROH. However, the Brazilian Karitiana and Pakistani Baluchi both show a large range for total lengths in ROH per individual. Outliers can be seen for the Senegalese Mandenka, the French and Japanese.

Many aspects of human genetic variation are patterned by our origin and subsequent dispersal out of Africa, including autosomal and Y chromosome genetic diversity [10], as well as estimates of expansion times and effective population size for Y chromosomes [13]. In a similar fashion, both the number (*r = *0.93, *p*≤0.0001) and sum (*r = *0.84, *p*≤0.0001) of ROH are strongly and significantly correlated with the overland distance from Addis Ababa. Breaking ROH down by size reveals that this pattern is driven by the shorter ROH (<2 Mb; *r = *0.93, *p*≤0.0001), more than the longer ROH (>16 Mb; *r = *0.52, *p = *0.0001). We repeated the analysis excluding Native Americans, who are extreme both in their distance from East Africa and ROH complement. The shorter ROH remained highly correlated with distance from Africa (*r = *0.82, *p*≤0.0001), but the longer ROH were no longer correlated (*r = *–0.27, *p = *0.07).

## Discussion

In common with recent studies of European-heritage populations [1], [4], [6], our analysis of this globally representative sample confirms that runs of homozygosity longer than 0.5 Mb are ubiquitous and frequent across all populations. Comparison of the number and sum length of ROH of various sizes to the mean inbreeding coefficient and effective population size indicate that ROH provide a valuable record of a population's demographic history. Moreover, ROH record the demographic history of the ancestors of the individual. In fact, in most populations individual ROH profiles cluster, particularly in terms of the number of ROH. Four classes of population can be distinguished on the basis of the length and number of ROH, which describe a demographic historical space. First the consanguineous populations from South and West Asia stand out in carrying significantly more very long ROH than the African and other Eurasian populations. Second the Oceanian populations are unique in having very large numbers of shorter ROH, but few long ROH, consistent with a reduced N_e_ in the past, but little inbreeding in recent times. Third the Native American populations have more short and long runs than any other population, indicating both ancient and recent parental relatedness. Fourth, the Europeans, East Asians and Africans show only the background level of shared ancestry relating to their continental N_e_.

The distribution of the shorter (0.5–2 Mb) ROH across the globe mirrors other aspects of human variation in reflecting our origin in sub-Saharan Africa and subsequent dispersals out of Africa, finally reaching Oceania and America. Analysis of ROH in three populations using 3.1 million markers revealed the same Africa, Europe, East Asia ranking [5]. The shortest category of ROH considered here (0.5–1 Mb) were inversely correlated with estimates of male N_e_ based on Y chromosome diversity. We note that the Y chromosome estimates of N_e_ are derived from independent data and thus avoid the circularity of comparing homozygosity with estimates of N_e_ derived from LD, which itself influences ROH. It must be remembered that the Y chromosome only records male history and furthermore represents only one realisation of the evolutionary process, so it will be interesting to compare to estimates of effective population size which also include mtDNA and/or whole genome variation. Longer ROH provide information on more recent ancestry, from population size and endogamy to recent inbreeding. However, unlike genetic diversity, linkage disequilibrium and other summary statistics of gene genealogies, ROH are an individual-level phenomenon and so provide a distinctive record of the demographic history of an individual's ancestors.

The increased numbers and so total length of ROH from 0.5–2 Mb in certain populations is likely to be the result of common extended haplotypes reaching high frequencies in these small, isolated communities, such that they are frequently inherited from both parents. Such patterns of haplotype sharing – among individuals or among parents of individuals – can also be described as correlations among alleles at different loci or LD. Because we are interested in the potential expression of recessive effects, we include ROH regardless of local patterns of LD; the small sample sizes available also prevent unbiased estimates of LD. These shorter ROH do not arise from inbreeding in recent generations and are common in all of the populations represented in the HGDP. In all populations shorter ROH make up the bulk of the homozygosity present. Even in the most inbred populations, the total length of the genome in shorter ROH (0. 5–2 Mb) is more than that in longer ROH (over 2 Mb). This point can be illustrated more dramatically by using a panel of 3 million SNPs, which enables the reliable detection of ROH as short as 100 kb. Using this panel, the total ROH length among Han Chinese is ∼510 Mb [5], compared with ∼130 Mb using a panel of ∼400,000 SNPs). Given that shorter ROH account for more of the total homozygosity even in the most inbred people, any effect of ROH on disease risk could also be mediated by these shorter runs, and not only by the long ROH arising from recent parental relatedness.

While there is a distribution of ROH number and length in all populations, this is very variable in consanguineous populations such as the South Asian Baluchi – not everyone has consanguineous parents – and as inheritance is a stochastic process, the outcome can vary widely even between siblings. South Asians living in Europe also show an increased variance in total ROH length [14]. Cryptically inbred outliers can be observed even in large predominantly urban populations, such as the French, Japanese and Mandenka. Such individuals plot to the right of their population in a graph of the number *vs.* sum length of ROH (e.g. Fig 3), as the relatively small number of very long ROH originating from their recent shared ancestry influence the sum of ROH more than the total number.

The comparison of the number and sum length of runs (e.g. Fig 3) is therefore a useful representation of the demographic history of an individual's ancestors. Individuals falling near the diagonal line carry a complement of ROH deriving from their continental N_e_, with the number of ROH being driven mostly by the more numerous shorter runs, which reflect more ancient demographic history. Africans have the lowest number of ROH, followed by South and West Asians, Europeans, East Asians, Oceanians and finally Native Americans. The distance along the sum of ROH axis from the diagonal differentiates individuals primarily in terms of their complement of long runs, and therefore recent inbreeding, with many south and west Asians and Native Americans undergoing a right shift (Fig 3 and File S1).

Individuals from particular populations stand somewhat apart from others of their continent due to a “right shift” (File S1), e.g. most of the very isolated Yakuts, and to a lesser degree some Sardinians, whilst the Maya have less homozygosity than the other Native Americans sampled, perhaps due to European admixture [9].

ROH, like other aspects of our genetic variation including genetic diversity and linkage disequilibrium, demonstrate a strong correlation with distance from East Africa. This pattern was driven mainly by the shorter ROH, consistent with their origin long enough ago to be influenced by serial founder events as humans spread across the globe. Longer ROH were less correlated with walking distance from Africa. When the analyses were repeated without Native Americans, to exclude the possibility of an increase in their homozygosity levels for reasons other than their distance from Africa, the shorter ROH remained highly correlated, but there was no significant relationship between distance from Africa and sum of ROH over 16 Mb in length. The lack of relationship for longer ROH is not surprising, given their very recent origins. The correlation with Out-of-Africa distances for shorter ROH show that patterns of identity-by-descent are structured by our migrations from Africa, not just identity-by-state observed through single SNP analyses of diversity.

Regions of extended homozygosity in the genome can also be a consequence of deletions along the genome. In this study it was not possible to check whether the observed ROH are the result of hemizygous deletions due to the fact that only the called genotyping data were available. However, based on previous studies, it would be reasonable to assume that ROH in this study are true homozygous tracts and not hemizygous deletions – apart from anything else many of the tracts observed here are much larger than typical copy number polymorphisms. Recent studies with access to fluorescent intensity data [1], [15], [16] reported that the observed extended regions of homozygosity are not the result of large deletion polymorphisms. This indicates that long homozygous regions are the result of a single ancestral haplotype being inherited from both parents, rather than the mark of copy number variation.

It is clear that a greater proportion of the genome in ROH will increase the individual risk of recessive Mendelian disease, but if many recessive factors are also involved in complex disease susceptibility, ROH may also confer risk for common diseases such as diabetes and heart disease. In line with this hypothesis, pedigree studies of hypertension and LDL cholesterol [17], quantitative genetic estimates of dominance variance for blood pressure, LDL, fasting insulin and measures of lung function [18] and analysis of genome-wide heterozygosity using microsatellites [19] all suggest that numerous recessive variants contribute to complex disease risk.

Long ROH are a neglected feature of our genome, which we have shown here to be universally common in human populations and to correlate well with demographic history. ROH are, however, only partially predictable from an individual's background (due to the stochastic nature of inheritance). As well as conferring susceptibility to recessive Mendelian diseases, ROH are also potentially an underappreciated risk factor for common complex diseases, given the evidence for a recessive component in many complex disease traits [18]; they will allow quantification of the risk arising from recessive genetic variants in different populations.

## Materials and Methods

The HGDP dataset includes populations from all continents with diverse demographic and cultural histories, from small isolated Amazonian groups to the Han Chinese. A number of populations have well documented preferences for consanguineous marriage [11]: Bedouin [20], Druze [21], Palestinians [22], Mozabite Berbers [23], Brahui [24], Baluchi [25], Makrani [26], Sindhi [27] and Pathan [28]. In some cases, population sample sizes are low, such that estimates inevitably have wide confidence intervals; however we have addressed this by analysing data at the continental as well as population level. The populations were grouped into seven distinct ‘continental’ groups (Europe, sub-Saharan Africa, America, Oceania, East Asia, Central/South Asia and West Asia). The HGDP dataset includes several individuals that are first or second degree relatives of others in the dataset. In this analysis all of the 1043 individuals were used as significant correlation in homozygosity is only expected for sibs, and even this is very variable [1].

Genotypes were available for 660,918 single nucleotide polymorphism markers (SNPs) from the Illumina 650Y product [10], [29]. Only SNPs on the 22 autosomes were included in this analysis (in total 644,258). SNPs located in the centromere regions were also removed, along with those with more than 10% missing genotypes. In addition, we excluded SNPs that failed an exact test of Hardy-Weinberg equilibrium in each population separately. The number of SNPs with *p*<0.0001 varied from 0 – 85, depending on the population; in total 735 SNPs were removed.

Ascertainment bias is a consequence of the small number of individuals in the SNP discovery panel and the bias in their geographic origins, so that the probability that a SNP is discovered is dependent on the allele frequency [30]. The Illumina arrays used in this study mostly contain tag SNPs derived from the HapMap populations: Yoruba from Nigeria, Han Chinese, Japanese and European Americans. SNPs represented in the Illumina array will therefore only represent common variants in those four populations and not SNPs specific to other regions of the world, or which are rarer in the ascertainment populations and more common in other places. The populations that are most distant genetically to the four HapMap populations will be most affected by this ascertainment bias, i.e. those from the Americas and Oceania, and indeed considerable numbers of SNPs were not polymorphic in these populations. This could inflate estimates of ROH length and frequency because a proportion of apparent ROH may in fact consist of consecutive non-polymorphic SNPs, which provide no information on identity by descent. To correct for this ascertainment bias, SNPs with frequencies less that 1% in any of the 7 continental regions were also removed, leaving a consensus panel of 415,130 SNPs to be used in the final analysis. Using different minor allele frequency thresholds up to 20% changed the absolute values of ROH statistics, but had little effect on the relative order of individuals or populations. As all ROH, long and short, ultimately arise through shared parental ancestry, recently or thousands of years ago, we did not account for linkage disequilibrium (LD) in the analyses. It is, however, unusual for LD to extend over 500 kb, the minimum ROH length considered here.

Runs of homozygosity were identified in PLINK [31], which takes a window of 5000 kb (50 SNPs) and slides it across the genome, determining homozygosity at each window. It allows for 1 heterozygous call per window and 5 missing calls. For each SNP, the proportion of homozygous windows that overlap that position is calculated and based on the threshold value of 0.05 (average for the SNPs in a segment), segments are called homozygous or not. The minimum length of a run of homozygosity was set to be 500 kb. This length was chosen as it approaches the limit of resolution given the SNP density of the array and also avoids counting short ROH where LD complicates matters. To ensure that locally low SNP density does not increase the length of an ROH, a required minimum density was set at 50 kb/SNP and the maximum gap between two consecutive SNPs in a tract was set at 100 kb.

Minimum SNP coverage in this dataset is 6.5 kb/SNP, so on average the minimum number of SNPs in the run will be 77. However, the density of SNPs is not consistent throughout the genome, and it is therefore important to ensure that there are enough SNPs to constitute a true ROH or homozygosity-by-descent. The minimum number of SNPs in a homozygous tract was set at 50 to make sure that the unobserved markers between the first and the last SNP in the run are also very likely to be homozygous.

Calculations were performed using SPSS and R software. A mixed linear model was used to check whether the values of the median, the 75th percentile and the number of ROH differed between the continents. The median and the 75^th^ percentile values of the ROH per individual were used to check for the differences in length of ROH. The 75^th^ percentile was used to test for the differences between longer ROH. The number of ROH was logarithmically transformed as it was not normally distributed. Due to the fact that the data are unbalanced (each continent contains different number of populations, and each population contains different number of individuals), restricted maximum likelihood (REML) estimation of variance components was used (implemented in R). In the model, continents were treated as a fixed effect and because each continent was subdivided into several populations, populations and individuals within the populations were treated as random effects. Chi squared tests were performed between each continent to determine whether there are significant differences between the proportions of individuals with ROH of different lengths (2–4 Mb, 4–8 Mb, 8–16 Mb, >16 Mb). Shorter length categories were not tested because all of the individuals from all populations have ROH shorter than this. Bonferroni correction for multiple testing (89 tests) was applied, and a significance threshold was set at *p = *0.00056. Individuals from West Asia (N = 176) and Central/South Asia (N = 207) were grouped together and tested against the combined group of Europe (N = 160) and East Asia (N = 235). The Mann-Whitney U test was used to test differences between the total mean lengths of ROH of the individuals between continental regions. Differences were tested for the mean length for each individual and four different cut-off lengths were used (>1.5 Mb, >3 Mb, >5 Mb, >10 Mb). Bonferroni correction for multiple testing (84 tests) was applied, and significance threshold was set at *p = *0.0006. Correlations between ROH measures and pedigree inbreeding (F_ped_) were calculated for 17 different populations for which pedigree-based mean inbreeding coefficients were available [11]. Where several F_ped_ estimates were available for one population, the mean of those estimates was used. For each individual, the F_ROH_ measure of homozygosity was calculated [1]. F_ROH_ was calculated by dividing the sum of ROH per individual by the total length covered by SNPs, excluding the centromeres (2,682.410 Mb).

As the rate of decay of LD varies across the genome, a threshold based on physical distance might not be appropriate in regions where haplotype blocks are unusually long or unusually short in outbred populations. To allow for this, we used the genetic map distance estimates from HapMap [5] (which are based on modelling haplotype structure as a mosaic of HapMap source haplotypes) to set different thresholds for defining ROH. Using thresholds from 0.04 to 5 cM, we recovered a perfectly correlated dataset. For example the proportion of ROH >4 Mb in length was correlated (*r = *0.9993, *p*<0.0001) with the proportion of ROH identified using a 5 cM threshold, while the sum of ROH >0.5 Mb was also perfectly correlated (*r = *0.9999, *p*<0.0001) with the sum of ROH called using a 0.04 cM threshold. Walking distances from Addis Ababa were taken from reference [10].

## Supporting Information

File S1 Supplementary tables and figures.
(0.28 MB PDF)
