# Supplementary material for: Genomic Runs of Homozygosity Record Population History and Consanguinity
Source: PLoS One. 2010 Nov 15;5(11):e13996. doi: 10.1371/journal.pone.0013996 (PMC2981575; doi:10.1371/journal.pone.0013996)
Supplement: File S1 — Supplementary tables and figures. (0.28 MB PDF) [file pone.0013996.s001.pdf]

ONLINE SUPPLEMENTARY TABLES AND FIGURES – KIRIN ET AL

Supplementary Table 1. Wald test statistic and significance levels for the linear mixed model.

Variable	Wald test	P value
Median	6.18	0.0001
75th percentile	6.72	<0.0001
Number of ROH	82.23	<0.0001

There is a significant difference between the number and the length of ROH between all the continents. The differences between the length of ROH are tested using the median and the 75th percentile values of ROH per individual within a continent.

Supplementary Table 2. Difference between the proportion of the individuals with ROH of different lengths between continental regions were tested by χ^2 test. P-values are given in the tables below. Differences were tested for four different length ranges (2-4 Mb, 4-8 Mb, 8-16 Mb, >16 Mb). Significant results are highlighted.

2-4Mb	**C/S America**	**Oceania**	**C/S Asia**	**W Asia**	**E Asia**	**Europe**
**Oceania**	0.5623					
**C/S Asia**	**0.0004**	0.0568				
**W Asia**	0.0015	0.0934	0.6824			
**E Asia**	**<0.0001**	0.0014	0.0008	**0.0002**		
**Europe**	**<0.0001**	0.0043	0.0176	0.0055	0.5353	
**Africa**	**<0.0001**	0.0129	0.1741	0.0718	0.1794	0.54
4-8 Mb	**C/S America**	**Oceania**	**C/S Asia**	**W Asia**	**E Asia**	**Europe**
**Oceania**	0.0887					
**C/S Asia**	**<0.0001**	0.8045				
**W Asia**	0.0575	0.7349	0.1058			
**E Asia**	**<0.0001**	**<0.0001**	**<0.0001**	**<0.0001**		
**Europe**	**<0.0001**	0.0009	**<0.0001**	**<0.0001**	0.4388	
**Africa**	**<0.0001**	0.0821	0.0136	**0.0001**	0.0014	0.0293
8-16 Mb	**C/S America**	**Oceania**	**C/S Asia**	**W Asia**	**E Asia**	**Europe**
**Oceania**	**<0.0001**					
**C/S Asia**	**<0.0001**	0.0820				
**W Asia**	0.00892	0.0073	0.0963			
**E Asia**	**<0.0001**	0.1685	**<0.0001**	**<0.0001**		
**Europe**	**<0.0001**	1	**<0.0001**	**<0.0001**	0.9300	
**Africa**	**<0.0001**	0.2540	**<0.0001**	**<0.0001**	0.1703	0.1965
>16 Mb	**C/S America**	**Oceania**	**C/S Asia**	**W Asia**	**E Asia**	**Europe**
**Oceania**	**0.0001**					
**C/S Asia**	**<0.0001**	0.0848				
**W Asia**	**<0.0001**	0.0784	0.9905			
**E Asia**	**<0.0001**	0.2663	**<0.0001**	**<0.0001**		
**Europe**	**<0.0001**	0.9772	**<0.0001**	**<0.0001**	0.2135	
**Africa**	**<0.0001**	0.9021	**0.0001**	**0.0001**	0.3664	0.8996

Supplementary Table 3. Difference between the total mean length of ROH of the individuals between continental regions were tested by Mann-Whitney test. P-values are given in the tables bellow. Differences were tested for the four different cut-off lengths (>1.5 Mb, >3 Mb, >5 Mb, >10 Mb). Significant results are highlighted.

>1.5 Mb	C/S America	Oceania	C/S Asia	W Asia	E Asia	Europe
**Oceania**	**<0.0001**					
**C/S Asia**	**<0.0001**	0.0653				
**W Asia**	**<0.0001**	0.1428	0.3143			
**E Asia**	**<0.0001**	**<0.0001**	**<0.0001**	**<0.0001**		
**Europe**	**<0.0001**	**<0.0001**	**<0.0001**	**<0.0001**	0.7742	
**Africa**	**<0.0001**	**<0.0001**	**<0.0001**	**<0.0001**	0.3209	0.1646
>3Mb	**C/S America**	**Oceania**	**C/S Asia**	**W Asia**	**E Asia**	**Europe**
**Oceania**	**<0.0001**					
**C/S Asia**	**<0.0001**	0.5727				
**W Asia**	**<0.0001**	0.1044	0.319			
**E Asia**	**<0.0001**	**<0.0001**	**<0.0001**	**<0.0001**		
**Europe**	**<0.0001**	**<0.0001**	**<0.0001**	**<0.0001**	0.1838	
**Africa**	**<0.0001**	0.0002	**<0.0001**[7]	**<0.0001**	0.0003	0.0069
>5 Mb	**C/S America**	**Oceania**	**C/S Asia**	**W Asia**	**E Asia**	**Europe**
**Oceania**	**<0.0001**					
**C/S Asia**	**<0.0001**	0.0214				
**W Asia**	**<0.0001**	0.0027	0.4291			
**E Asia**	**<0.0001**	0.0010	**<0.0001**	**<0.0001**		
**Europe**	**<0.0001**	0.0001	**<0.0001**	**<0.0001**	0.2578	
**Africa**	**<0.0001**	0.1484	**<0.0001**	**<0.0001**	0.0057	0.0005
>10 Mb	**C/S America**	**Oceania**	**C/S Asia**	**W Asia**	**E Asia**	**Europe**
**Oceania**	**<0.0001**					
**C/S Asia**	**<0.0001**	0.0038				
**W Asia**	**<0.0001**	0.0002	0.2287			
**E Asia**	**<<0.0001**	0.1642	**<0.0001**	**<0.0001**		
**Europe**	**<0.0001**	0.5267	**<0.0001**	**<0.0001**	0.2952	
**Africa**	**<0.0001**	0.7235	**<0.0001**	**<0.0001**	0.1548	0.6996

Supplementary Table 4. Central/South Asian and West Asian populations are grouped together as are the European and East Asian populations. Differences between these two new groups were tested by χ^2. P values are given in the table. Differences were tested for four different ROH length categories (2-4 Mb, 4-8 Mb,8-16 Mb, >16 Mb).

	2-4 Mb	4-8 Mb	8-16 Mb	>16
(Central/South Asia + West Asia) - (East Asia + Europe)	**<0.0001**	**<0.0001**	**<0.0001**	**<0.0001**

Supplementary Table 5. Proportion of the genome (%) covered in runs of homozygosity above the give threshold for the probability of an extended haplotype. Results are given as an average value for an individual in a given population.

Population	no threshold	>0.04 cM	>0.05 cM	>0.06 cM	>0.1cM	>0.5 cM	>1 cM	>2 cM	>5 cM
Balochi	7.9120	7.6659	7.6647	7.6611	7.5798	5.6639	4.9527	4.4290	3.0969
Brahui	5.9733	5.7482	5.7408	5.7358	5.6641	3.6563	2.9852	2.5265	1.6578
Burusho	4.0931	3.9653	3.9608	3.9569	3.8747	1.9775	1.3083	1.0519	0.6459
Hazara	4.4820	4.3073	4.3013	4.2887	4.2094	2.1729	1.4771	1.2852	0.8493
Kalash	8.1577	7.8841	7.8825	7.8805	7.7913	5.5992	4.5696	3.5510	1.6361
Makrani	7.0363	6.7998	6.7973	6.7937	6.7227	4.8723	4.1795	3.7894	2.6708
Pathan	5.7090	5.4922	5.4884	5.4848	5.4086	3.4883	2.8205	2.5902	1.8950
Sindhi	5.4996	5.2988	5.2976	5.2942	5.2145	3.3272	2.7021	2.3820	1.6087
Uygur	3.0459	2.9457	2.9437	2.9348	2.8666	0.9691	0.4169	0.3043	0.2545
Bantu	1.8139	1.7698	1.7698	1.7673	1.7228	1.2066	0.8140	0.5032	0.2162
Biaka Pygmies	2.8909	2.7718	2.7706	2.7679	2.7301	2.2412	1.8371	1.1928	0.3497
Mandenka	1.8155	1.7559	1.7546	1.7521	1.7208	0.9700	0.6272	0.5205	0.3731
Mbuti Pygmies	3.5846	3.4658	3.4658	3.4571	3.4085	2.4566	1.7347	0.9791	0.4231
San	4.0096	3.8698	3.8698	3.8698	3.8049	2.9209	2.2495	1.7100	0.8023
Yoruba	1.6059	1.5335	1.5309	1.5309	1.4763	0.7508	0.3255	0.1380	0.0686
Colombian	15.0126	14.5989	14.5989	14.5989	14.5020	10.4331	6.2256	3.9881	2.7625
Karitiana	20.5416	19.8365	19.8357	19.8316	19.7575	15.5160	10.8608	8.1562	5.3507
Maya	9.3244	8.9956	8.9937	8.9849	8.9041	5.1461	1.6904	0.4815	0.3055
Pima	16.1817	15.6636	15.6625	15.6524	15.5742	11.8951	7.9883	5.9443	4.0999
Surui	25.0511	24.1767	24.1767	24.1767	24.1013	19.9967	15.4295	12.6948	9.1760
Adygei	4.0152	3.8625	3.8596	3.8449	3.7752	1.5821	0.6906	0.4771	0.2978
Basque	5.4703	5.2825	5.2756	5.2688	5.1813	2.6805	1.4127	0.8059	0.2558
French	4.2608	4.0796	4.0786	4.0702	3.9923	1.5120	0.4938	0.2866	0.2054
Italia	4.6552	4.5177	4.5177	4.5128	4.4158	1.9931	1.0776	0.8631	0.6644
Orcadian	4.4886	4.3373	4.3349	4.3257	4.2393	1.8114	0.7318	0.4653	0.2006
Russian	4.1194	3.9581	3.9562	3.9529	3.8878	1.4931	0.4183	0.1541	0.0250
Sardinian	5.3196	5.0972	5.0930	5.0849	5.0127	2.4353	1.1416	0.7207	0.3021
Tuscan	3.7727	3.5702	3.5663	3.5632	3.4880	1.3146	0.3246	0.1493	0.1017
Melanesian	10.7464	10.3808	10.3724	10.3699	10.2822	6.3807	3.0053	1.1444	0.3299
Papuan	12.7762	12.4377	12.4377	12.4308	12.3563	7.8225	3.7670	1.8569	0.7512
Cambodian	4.5366	4.3916	4.3882	4.3833	4.2978	1.5612	0.4354	0.1372	0.0432
Dai	5.3845	5.1609	5.1532	5.1507	5.0698	2.0485	0.6327	0.3752	0.2018
Daur	4.9901	4.7992	4.7960	4.7901	4.7053	1.9639	0.6466	0.3299	0.1163
Han	4.7277	4.5792	4.5753	4.5690	4.4733	1.5091	0.2242	0.0238	0.0036
Hezhen	5.5268	5.3364	5.3271	5.3198	5.1979	2.2103	0.8630	0.5866	0.3948
Japanese	5.5847	5.3686	5.3654	5.3537	5.2692	2.0844	0.6189	0.2583	0.1790
Lahu	6.8120	6.6167	6.6167	6.6019	6.5174	3.7878	2.3996	1.5518	0.5948
Miaozu	5.6755	5.4856	5.4799	5.4726	5.3717	2.5089	1.1220	0.7024	0.2805
Mongol	4.2446	4.1162	4.1162	4.1107	3.9982	1.2844	0.1895	0.0507	0.0100
Naxi	5.3733	5.1969	5.1927	5.1873	5.0623	2.1333	0.7011	0.3403	0.1298
Oroqen	5.5926	5.4553	5.4535	5.4507	5.3790	2.5951	1.0848	0.5533	0.1628
She	5.7768	5.5653	5.5653	5.5600	5.4739	2.5530	1.2760	0.9034	0.4840
Tu	4.2919	4.1056	4.0984	4.0937	3.9983	1.2021	0.2554	0.1312	0.0882
Tujia	5.6475	5.4176	5.4176	5.4103	5.3193	2.3356	1.1694	0.9925	0.7915
Xibo	4.3012	4.1445	4.1393	4.1297	4.0434	1.3105	0.1579	0.0244	0.0244
Yakut	6.5391	6.3441	6.3399	6.3366	6.2575	3.5958	2.2157	1.5396	0.5800
Yizu	5.4255	5.2007	5.1988	5.1968	5.1067	2.1666	0.8097	0.4578	0.1401
Bedouin	7.1589	6.9066	6.9040	6.8997	6.8307	4.9140	4.0001	3.3658	2.1784
Druze	6.6461	6.4484	6.4471	6.4393	6.3530	4.1369	3.3476	3.0531	2.1589
Mozabite	4.7206	4.5784	4.5784	4.5761	4.4992	2.9841	2.2837	1.7489	0.8059

Supplementary Figure. Individual patterns of long runs of homozygosity. The number of runs of homozygosity compared to the total length in ROH for each individual in the HGDP by population for each continental region.

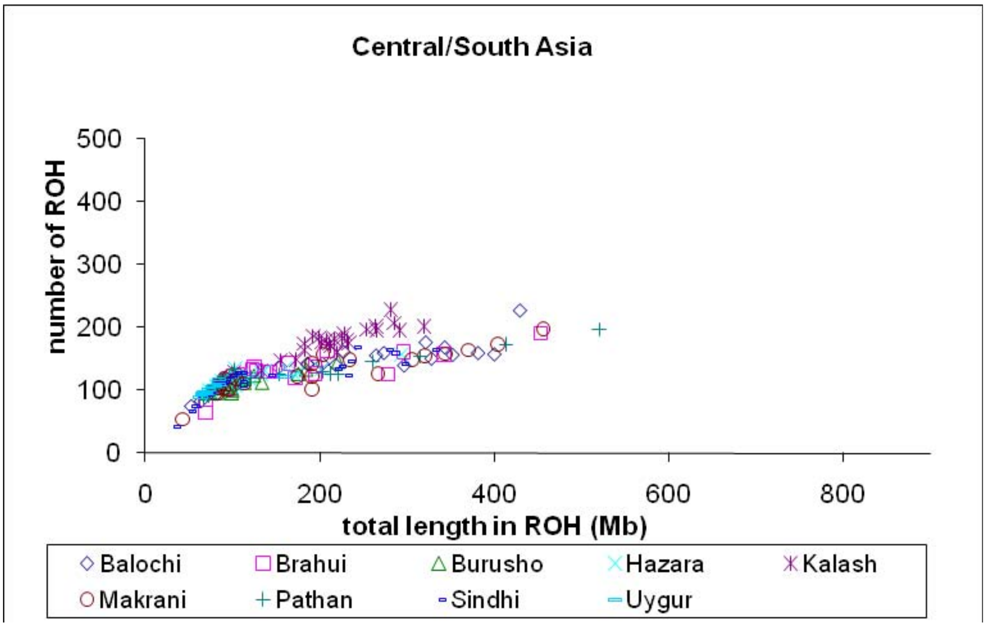

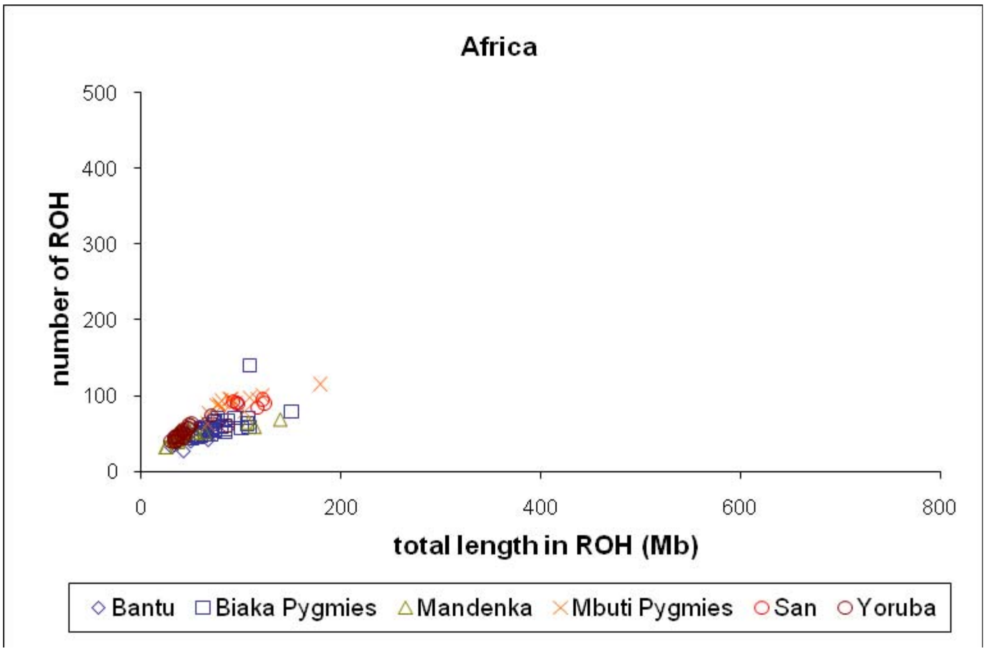

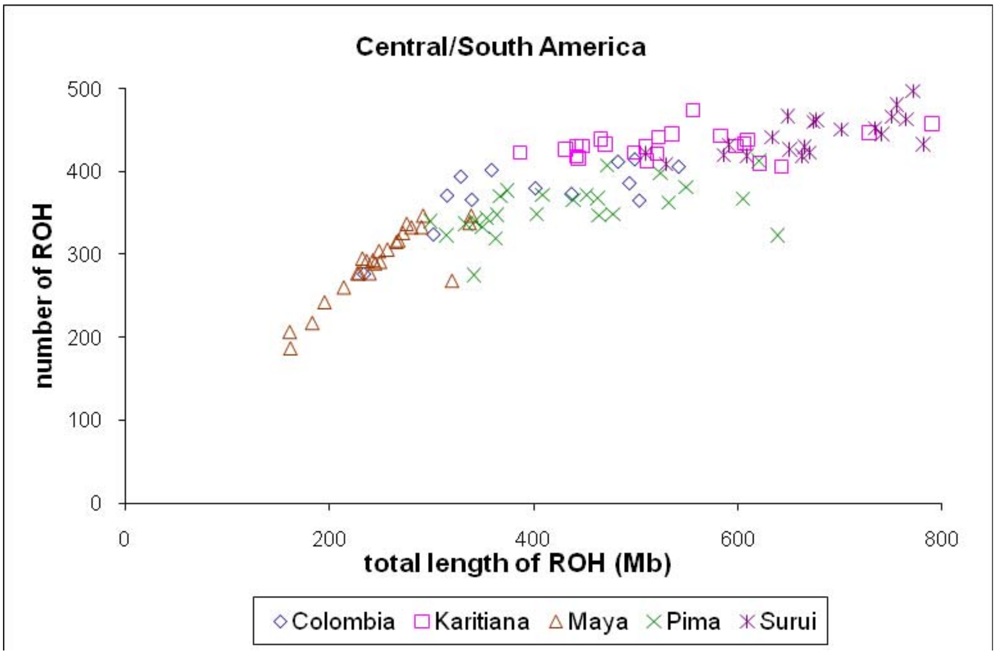
Central/South America
number of ROH
total length of ROH (Mb)
0
100
200
300
400
500
0
200
400
600
800
Colombia
Karitiana
Maya
Pima
Surui

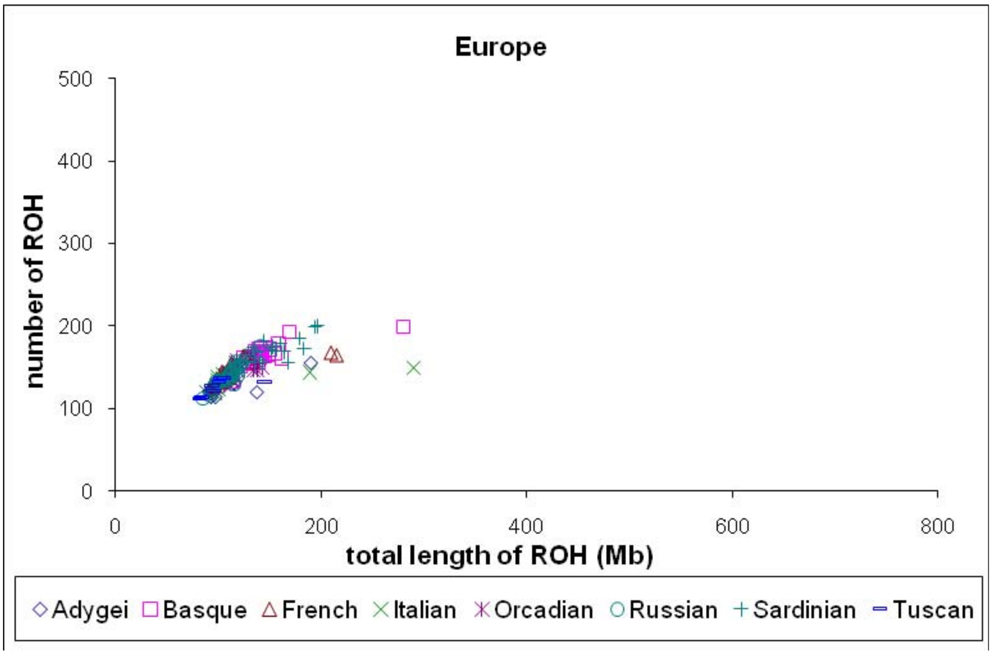
Europe
number of ROH
total length of ROH (Mb)
0
100
200
300
400
500
0
200
400
600
800
Adygei
Basque
French
Italian
Orcadian
Russian
Sardinian
Tuscan

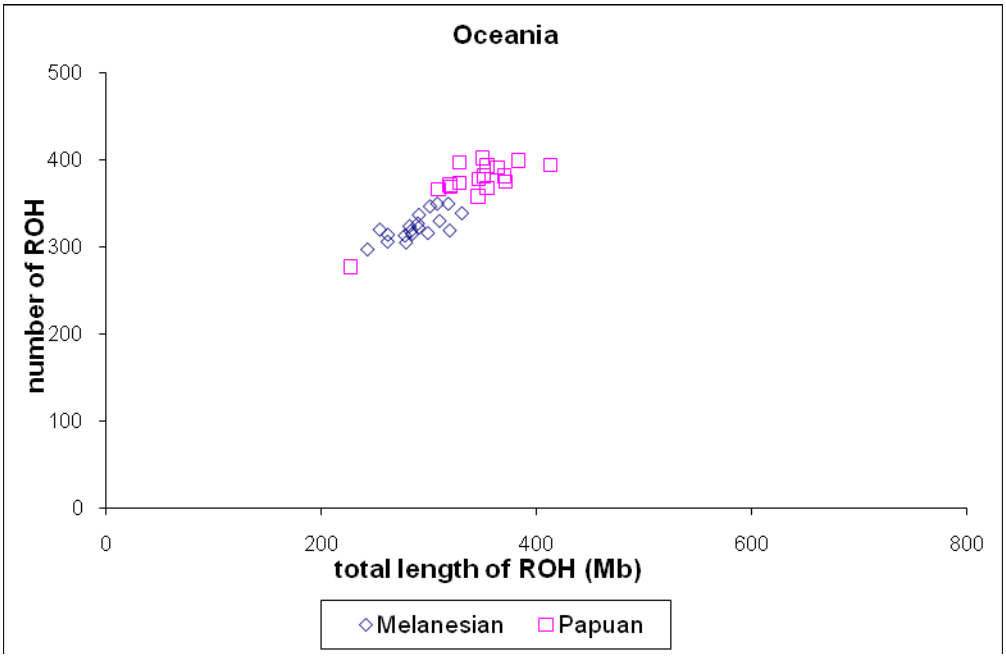
Oceania
number of ROH
total length of ROH (Mb)
Melanesian
Papuan

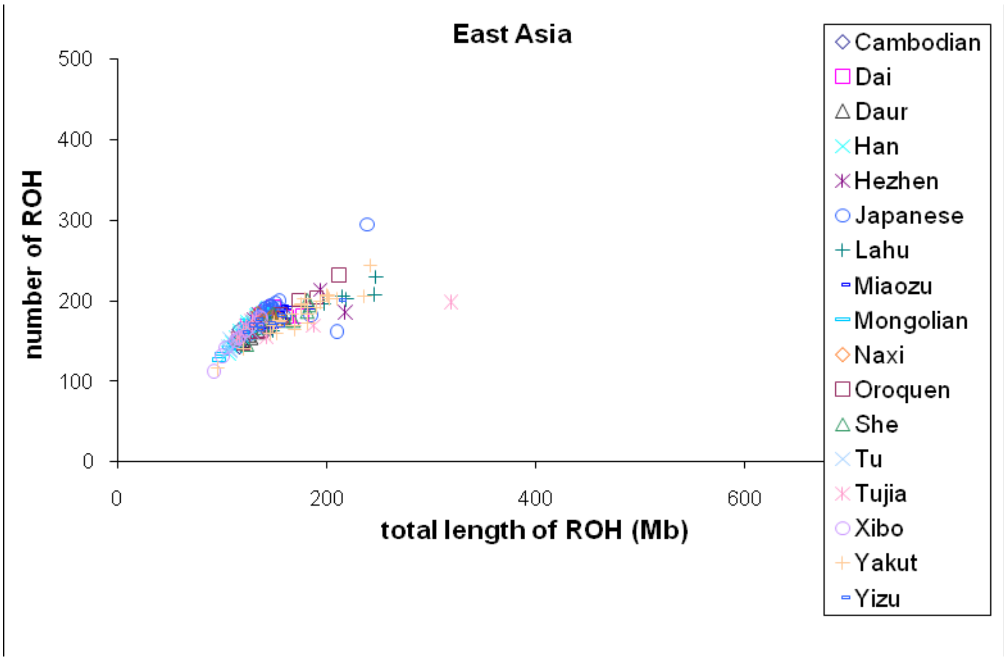
East Asia
number of ROH
total length of ROH (Mb)
Cambodian
Dai
Daur
Han
Hezhen
Japanese
Lahu
Miaozu
Mongolian
Naxi
Oroquen
She
Tu
Tujia
Xibo
Yakut
Yizu

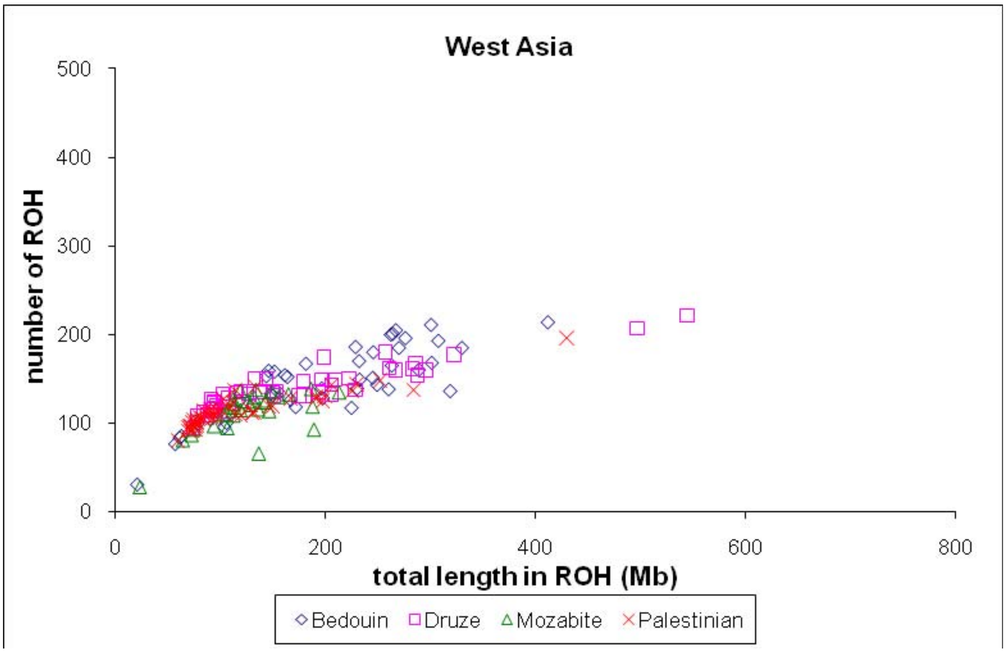
West Asia
500
400
300
200
100
0
number of ROH
0
200
400
600
800
total length in ROH (Mb)
Bedouin
Druze
Mozabite
Palestinian
